# DJ-1 links muscle ROS production with metabolic reprogramming and systemic energy homeostasis in mice

**DOI:** 10.1038/ncomms8415

**Published:** 2015-06-16

**Authors:** Sally Yu Shi, Shun-Yan Lu, Tharini Sivasubramaniyam, Xavier S. Revelo, Erica P. Cai, Cynthia T. Luk, Stephanie A. Schroer, Prital Patel, Raymond H. Kim, Eric Bombardier, Joe Quadrilatero, A. Russell Tupling, Tak W. Mak, Daniel A. Winer, Minna Woo

**Affiliations:** 1 Toronto General Research Institute, University Health Network, MaRS Centre, TMDT, 101 College Street, 10th floor, Room 10-363, Toronto, Ontario M5G 1L7, Canada; 2 Institute of Medical Science, University of Toronto, Toronto, Ontario M5G 2M9, Canada; 3 The Campbell Family Institute for Breast Cancer Research, Ontario Cancer Institute, University Health Network, Toronto, Ontario M5G 2C1, Canada; 4 Department of Kinesiology, University of Waterloo, Waterloo, Ontario N2L 3G1, Canada; 5 Division of Endocrinology and Metabolism, Department of Medicine, University Health Network, University of Toronto, Toronto, Ontario M5G 2C4, Canada

## Abstract

Reactive oxygen species (ROS) have been linked to a wide variety of pathologies, including obesity and diabetes, but ROS also act as endogenous signalling molecules, regulating numerous biological processes. DJ-1 is one of the most evolutionarily conserved proteins across species, and mutations in DJ-1 have been linked to some cases of Parkinson's disease. Here we show that DJ-1 maintains cellular metabolic homeostasis via modulating ROS levels in murine skeletal muscles, revealing a role of DJ-1 in maintaining efficient fuel utilization. We demonstrate that, in the absence of DJ-1, ROS uncouple mitochondrial respiration and activate AMP-activated protein kinase, which triggers Warburg-like metabolic reprogramming in muscle cells. Accordingly, DJ-1 knockout mice exhibit higher energy expenditure and are protected from obesity, insulin resistance and diabetes in the setting of fuel surplus. Our data suggest that promoting mitochondrial uncoupling may be a potential strategy for the treatment of obesity-associated metabolic disorders.

## Abstract (web summary)

The protein DJ-1 is known to have antioxidant effects in cells. Here, the authors reveal that DJ-1 has a role in coupling mitochondrial respiration in skeletal muscles of mice, and show that absence of DJ-1 increases energy expenditure and protects mice from diet-induced obesity.

## Graphical abstract

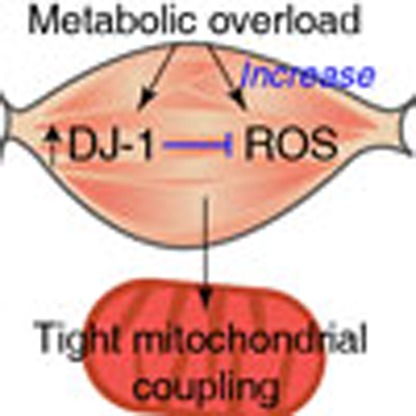

A fundamental physiological process in all living cells involves harnessing the energy stored in fuel substrates in an efficient manner for ATP generation. Thus living organisms have evolved a tightly regulated system to guard against any disruption in the metabolic pathways of fuel substrates^1^. During periods of famine, the efficient use and storage of energy confers a survival advantage^2^. However, in the modern world of sedentary lifestyles and easy access to calorie-dense foods, efficient fuel utilization often results in a surplus of energy, which is stored in the adipose tissue, thereby leading to the development of obesity and associated complications^3^.

Inefficient mitochondrial respiration generates reactive oxygen species (ROS), whose overproduction is thought to underlie a myriad of disorders including obesity-associated insulin resistance and type 2 diabetes. However, a growing body of evidence suggests that ROS act as signalling molecules and are required for maintaining physiological homeostasis^4^. For example, interventions that promote health and lifespan such as caloric restriction and physical exercise stimulate generation of free radicals in the mitochondria^5^, whereas antioxidant supplementation to attenuate ROS fails to demonstrate any beneficial effects in most of the large-scale intervention trials^6^,^7^,^8^,^9^. Indeed, antioxidant supplementation has been shown to counteract the insulin-sensitizing effects of exercise training in humans^10^.

ROS, in particular H_2_O_2_, promote insulin signalling by reversible oxidation and inhibition of protein tyrosine phosphatases (PTPs) such as PTP1B or phosphatase and tensin homologue (PTEN), thereby promoting insulin receptor autophosphorylation and phosphoinositide 3-kinase signalling, respectively^11^,^12^. In addition, mitochondria produce ROS in response to various stress signals, which lead to transcriptional changes in the nucleus in a process known as the retrograde response^13^. This activates cellular adaptive mechanisms that confer stress resistance and promote health and lifespan^14^,^15^. Whether ROS mediate the adaptive response to metabolic stress, and the specific molecular mechanisms responsible for ROS-induced health benefits are not completely understood.

DJ-1 is a highly conserved, ubiquitously expressed protein with homologues found in distant organisms including yeast and even bacteria^16^. DJ-1 is involved in the regulation of oxidative stress by directly quenching ROS upon oxidative modification of a conserved cysteine residue^17^ or by stabilizing the master regulator of antioxidant transcription, nuclear factor erythroid-related factor 2 (NRF2)^18^. Mutations in the gene encoding *DJ1* (also known as *PARK7*) are associated with early-onset recessive familial Parkinsonism^19^. As such, overexpression of *Dj1* protected neurons against oxidative stress-induced cell death^20^,^21^, whereas *Dj1* null mice exhibit increased susceptibility to a variety of oxidative insults^22^,^23^,^24^.

Despite the pro-survival and protective role of DJ-1 in neurons, an inactivating mutation in *dj-1β*, one of two DJ-1 orthologs in *Drosophila melanogaster*, was recently shown to increase mitochondrial H_2_O_2_ production and prolong lifespan under conditions of moderate oxidative stress^25^. These findings are consistent with the health-promoting role of ROS. Whether DJ-1 exerts a similar function in higher organisms via regulation of ROS is unknown.

In this study, we sought to examine the *in vivo* role of DJ-1, particularly in the context of chronic metabolic stress. We show that DJ-1 acts to promote efficient fuel utilization in the skeletal muscle. Elevated ROS induced by DJ-1 deficiency uncouple mitochondrial respiration and trigger Warburg-like metabolic reprogramming with activation of AMP-activated protein kinase (AMPK) and induction of glycolysis. These metabolic effects together increase energy expenditure in the skeletal muscle and confer resistance to obesity and diabetes in the setting of fuel surplus. Thus, our work identifies a novel metabolic role of the antioxidant protein DJ-1 and uncovers an important mechanism by which ROS confer health benefits.

## Results

### DJ-1 regulates ROS levels in skeletal muscle

**Figure 1:**
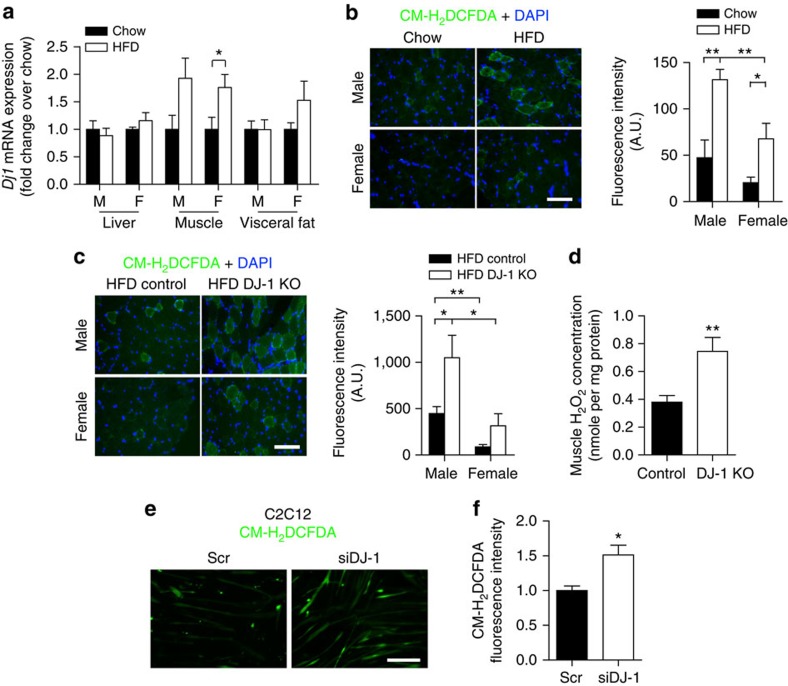
DJ-1 modulates ROS levels in mouse skeletal muscle. (**a**) mRNA levels of *Dj1* measured by quantitative RT–PCR in liver, quadriceps and perigonadal adipose tissue from C57BL/6 mice maintained on standard chow or fed a HFD for 3 months starting at 2 months of age (*n*=4–6 per group). F, females; M, males. (**b**,**c**) Representative micrographs of quadriceps cross-sections showing ROS levels assessed using CM-H_2_DCFDA and quantification of fluorescence intensity in (**b**) chow- and HFD-fed C57BL/6 mice and (**c**) HFD-fed control and DJ-1 KO mice (*n*=4–6 per group). Scale bar, 80 μm. (**d**) H_2_O_2_ levels measured using the Amplex Red reagent in quadriceps tissue from HFD-fed female mice and normalized to sample protein content (*n*=7 per group). (**e**) Representative micrographs showing ROS levels assessed using CM-H_2_DCFDA in C2C12 myotubes. Scr, scramble siRNA. Scale bar, 80 μm. (**f**) ROS levels assessed using CM-H_2_DCFDA in C2C12 myotubes measured using a fluorescence microplate reader. Results are presented as fold change relative to the scramble group from three independent experiments in triplicate. Results are presented as mean±s.e.m. according to the two-tailed unpaired Student's *t*-test. *P<0.05; **P<0.01.

Obesity-associated insulin resistance is often accompanied by oxidative stress with ROS accumulation in metabolic tissues^26^. Given its antioxidant role, we hypothesized that DJ-1 in metabolic tissues may modulate obesity-related ROS production. We first tested regulation of *Dj1* (also known as *Park7*) expression in C57BL/6 mice rendered obese and insulin resistant by prolonged high fat diet (HFD) feeding for 3 months. As shown in Fig. 1a, HFD upregulated *Dj1* messenger RNA (mRNA) in skeletal muscle, but not in liver or visceral adipose tissue. Closer examination of different muscles revealed an increase in *Dj1* in oxidative soleus muscle fibres but not in glycolytic extensor digitorum longus fibres following HFD (Supplementary Fig. 1a). In association with *Dj1* upregulation, we observed ROS accumulation in mouse skeletal muscle in response to HFD, with male mice displaying higher ROS levels under both chow and HFD conditions compared with female counterparts (Fig. 1b and Supplementary Fig. 1b). In line with its antioxidant role, DJ-1 deficiency led to a further increase in muscle ROS levels in HFD-fed mice (Fig. 1c,d and Supplementary Fig. 1c). This increase in ROS was due to a cell-autonomous role of DJ-1 in the skeletal muscle, as its knockdown in the mouse myoblast cell line C2C12 cells was sufficient to elevate intracellular ROS concentration (Fig. 1e,f and Supplementary Fig. 2). Together, these results suggest that DJ-1 modulates HFD-induced ROS production particularly in the skeletal muscle.

**Figure 2:**
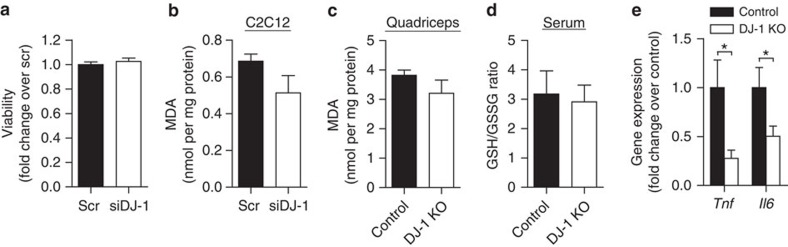
DJ-1 deficiency does not induce overt oxidative stress in muscle. (**a**) About 96 h after differentiation induction, C2C12 cell survival was assessed using an MTT assay. Results are from two independent experiments in five replicates. (**b,c**) Malondialdehyde (MDA) levels measured using a thiobarbituric acid reactive substances (TBARS) assay kit in (**b**) C2C12 myotubes after *Dj1* knockdown (*n*=3 per group) and (**c**) quadriceps tissue from female mice fed a HFD for 3 months starting at 2 months of age (*n*=4 per group). Results are normalized to sample protein content. (**d**) Reduced glutathione (GSH) to oxidized glutathione (GSSG) ratio in serum from HFD-fed female mice (*n*=7 for control and 8 for DJ-1 KO). (**e**) mRNA expression of inflammatory cytokines in quadriceps from HFD-fed female mice measured by quantitative RT–PCR (*n*=9 per group). Results are presented as mean±s.e.m. according to the two-tailed unpaired Student's *t*-test. *P<0.05.

Surprisingly, in contrast to pancreatic β-cells in which DJ-1 deficiency accelerated basal and H_2_O_2_-induced cell death and attenuated glucose-stimulated insulin secretion^27^,^28^ (Supplementary Fig. 3), *Dj1* knockdown in C2C12 myotubes had no effect on cellular viability despite elevated ROS levels (Fig. 2a). Furthermore, DJ-1-deficient cells and tissues showed comparable levels of malondialdehyde (MDA), a marker for ROS-dependent lipid peroxidation, as their respective controls (Fig. 2b,c and Supplementary Fig. 4a,b). Consistent with this, transcription of antioxidant enzymes or genes upregulated under oxidative stress conditions was not affected by DJ-1 deficiency in muscle tissue (Supplementary Fig. 4c). Circulating reduced to oxidized glutathione ratio (GSH/GSSG) and H_2_O_2_ level were also similar in DJ-1 knockout (KO) mice and control littermates (Fig. 2d and Supplementary Fig. 4d). Taken together, these results suggest that muscle ROS accumulation in the setting of DJ-1 deficiency did not induce overt oxidative stress or damage. In fact, while increased ROS are commonly linked to activation of a proinflammatory response^29^, DJ-1 KO mice showed lower *Tnf* and *Il6* expression in their skeletal muscle (Fig. 2e), indicating attenuated inflammation.

### DJ-1 deficiency activates glycolysis in muscle via ROS

**Figure 3:**
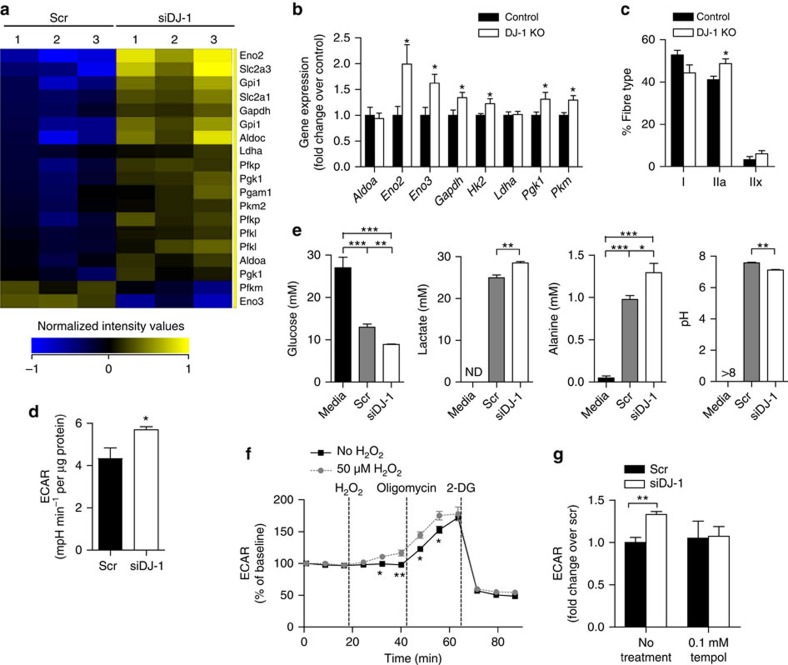
DJ-1 deficiency activates glycolysis in the skeletal muscle via ROS. (**a**) Heat map representation of normalized signal intensity values for genes involved in glycolysis with a *P* value<0.05. Gene expression was analysed by microarray in C2C12 myotubes after *Dj1* knockdown. Statistical significance was calculated using moderated student *t*-test followed by Benjamini–Hochberg false discovery rate correction in GeneSpring GX12.6 software. (**b**) mRNA expression of genes involved in glycolysis measured by quantitative RT–PCR in quadriceps from female mice fed a HFD for 3 months starting at 2 months of age (*n*=4–8 per group). (**c**) Fibre type distribution of soleus muscle from HFD-fed female mice (*n*=5 for control and 4 for DJ-1 KO). (**d**) Basal ECAR in C2C12 myotubes measured using the Seahorse flux analyzer and normalized to protein content (*n*=6 per group). Experiments were repeated at least three times. (**e**) Glucose, lactate, alanine and pH level in conditioned media from C2C12 myotubes (*n*=3 per group). ND, not detected. pH was >8 for the media group and was beyond the detection limit of the assay. (**f**) ECAR in C2C12 myotubes in response to 50 μM H_2_O_2,_ 1 μM oligomycin and 100 mM 2-deoxyglucose (2-DG) (*n*=3 per group). (**g**) Basal ECAR in myotubes treated with 0.1 mM tempol (*n*=3 per group). Results are presented as mean±s.e.m. according to the two-tailed unpaired Student's *t*-test for **b**–**d**,**f**,**g**, and one-way analysis of variance followed by Tukey's *post-hoc* test for (**e**). *P<0.05; **P<0.01; ***P<0.001.

We next examined the effect of muscle ROS accumulation at the transcription level and analysed gene expression by microarray in C2C12 myotubes. Intriguingly, DJ-1-deficient cells exhibited a general upregulation of genes involved in glycolysis (Fig. 3a). This was confirmed by quantitative reverse transcription (RT)–PCR analysis both in myotubes and *in vivo* in muscle tissue (Fig. 3b and Supplementary Fig. 5a). In line with a shift towards glycolytic metabolism, soleus muscle from DJ-1 KO mice showed a shift from type I slow-twitch oxidative fibres to type IIa fast-twitch oxidative and type IIx fast-twitch glycolytic fibres, suggesting muscle adaptation to enhance glycolytic capacity (Fig. 3c). Furthermore, DJ-1-deficient cells exhibited a higher extracellular acidification rate (ECAR) as measured by a Seahorse XF24 Analyzer, indicating increased glycolytic flux (Fig. 3d). Consistent with enhanced glycolysis and consequently increased lactate production, conditioned media from *Dj1* knockdown myotubes showed lower glucose concentration, higher lactate and higher alanine concentration with a more acidic pH (Fig. 3e). Metabolism of glutamine, another important oxidative fuel, did not seem to be affected by DJ-1 deficiency (Supplementary Fig. 5b).

ROS are known to activate the glycolytic programme particularly in cancer cells^30^ through stabilization of hypoxia inducible factor (HIF) 1α^31^. In this context, glycolysis is proposed to generate a chemically reduced milieu associated with inhibition of ROS-induced damage and enhanced antioxidant defense^32^. Indeed, addition of H_2_O_2_ induced a rapid increase in ECAR in C2C12 myotubes (Fig. 3f), whereas treatment of myotubes with the superoxide dismutase-mimetic and antioxidant tempol completely abolished the increase in ECAR induced by *Dj1* knockdown (Fig. 3g), indicating that ROS can mediate glycolytic activation also in metabolic tissues. However, co-transfection with HIF1α short interfering RNA (siRNA) had no significant effect on ECAR in *Dj1* knockdown myotubes (Supplementary Fig. 5c), suggesting that glycolytic induction in the setting of DJ-1 disruption was independent of the HIF1α pathway.

### DJ-1 deficiency induces mitochondrial uncoupling via ROS

**Figure 4:**
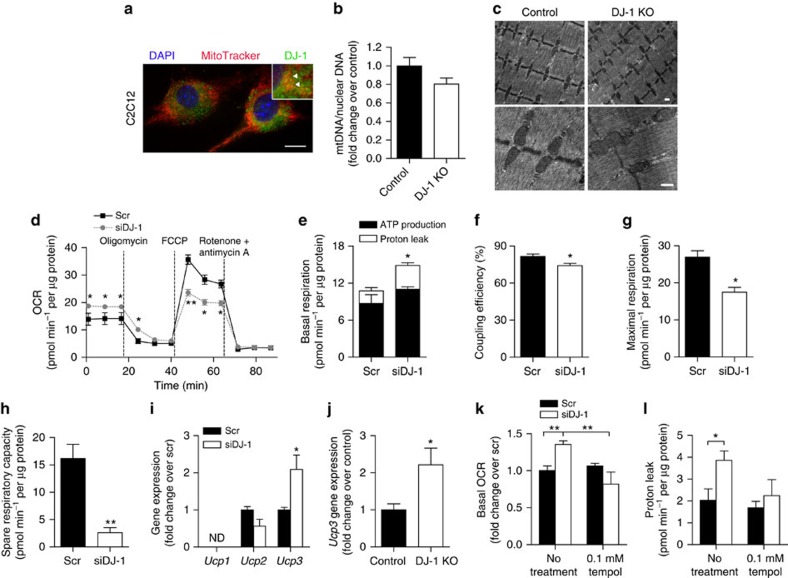
DJ-1 deficiency induces muscle mitochondrial uncoupling via ROS. (**a**) Representative micrograph of C2C12 myoblasts stained with MitoTracker and antibody against DJ-1. Scale bar, 10 μm. Inset: higher magnification image. Arrowhead, co-localization of DJ-1 with MitoTracker. (**b**) mtDNA copy number calculated as the ratio of *Cox2* to *Ppia* levels measured by real-time quantitative PCR in quadriceps tissue. Female mice were fed a HFD for 3 months starting at 2 months of age (*n*=5 per group). (**c**) Transmission electron microscopy images from quadriceps tissue of HFD-fed female mice. Scale bar, 500 nm. (**d**) OCR in C2C12 myotubes measured using the Seahorse flux analyzer in response to 1 μM rotenone, 0.5 μM FCCP, and 1 μM rotenone and antimycin A (*n*=3 per group). Experiments were repeated at least three times. (**e–h**) ATP production, proton leak, coupling efficiency, maximal respiration and spare respiratory capacity calculated from (**d**). (**i,j**) mRNA expression of uncoupling proteins measured by quantitative RT–PCR in (**i**) C2C12 myotubes (*n*=3–6 per group) and (**j**) quadriceps tissue of HFD-fed female mice (*n*=6 per group). ND, not detected. (**k**) Basal OCR and (**l**) proton leak in myotubes treated with 0.1 mM tempol (*n*=3 per group). Results are presented as mean±s.e.m. according to the two-tailed unpaired Student's *t*-test. *P<0.05; **P<0.01.

We therefore investigated alternative mechanisms mediating ROS-induced metabolic switch in muscle cells. DJ-1 acts to maintain mitochondrial dynamics^33^,^34^ and its disruption was shown to result in reduced mitochondrial transmembrane potential^35^. Consistent with a potential role of DJ-1 in mitochondrial homeostasis, we observed mitochondrial localization of a small fraction of DJ-1 protein in C2C12 cells (Fig. 4a). Mitochondrial content was not affected by DJ-1 deficiency, as indicated by no change in mitochondrial DNA (mtDNA) copy number and expression of genes involved in mitochondrial biogenesis (Fig. 4b and Supplementary Fig. 6a,b). Using transmission electron microscopy, we also found no apparent alteration in mitochondrial morphology or ultrastructure in DJ-1 KO mice (Fig. 4c). Next, to assess mitochondrial function, we analysed cellular bioenergetic profile using the Seahorse XF24 Analyzer (Fig. 4d). As shown in Fig. 4e, DJ-1-deficient cells had an increase in basal oxygen consumption rate (OCR) resulting mostly from a twofold elevation in proton leak, with mitochondrial ATP generation not significantly changed, leading to a reduction in coupling efficiency (Fig. 4f). Furthermore, addition of an exogenous uncoupler carbonyl cyanide-4-(trifluoromethoxy)phenylhydrazone (FCCP) increased OCR to a higher level in myotubes transfected with scramble siRNA than in *Dj1* knockdown cells (Fig. 4d). As such, DJ-1-deficient cells showed lower maximal respiration and spare respiratory capacity (Fig. 4g,h), suggesting that DJ-1 disruption reduces maximal respiration and/or uncouples the electron transport chain. Consistent with increased proton leak and reduced coupling efficiency, *Dj1* knockdown cells and DJ–1-deficient muscle tissue showed higher mRNA level of uncoupling protein 3 (*Ucp3*) (Fig. 4i,j), which can be activated by ROS^36^. This increase in mitochondrial uncoupling was dependent on ROS, as treatment with the antioxidant tempol abolished the increase in proton leak, leading to an attenuation in basal OCR in *Dj1* knockdown myotubes (Fig. 4k,l). Interestingly, despite induction of mitochondrial uncoupling, ATP concentration in muscle tissue was similar between DJ-1 KO mice and control littermates (Supplementary Fig. 6c), whereas *Dj1* knockdown in C2C12 myotubes led to an increase in cellular ATP levels (Supplementary Fig. 6d).

### AMPK mediates glycolytic induction in DJ-1 deficiency

**Figure 5:**
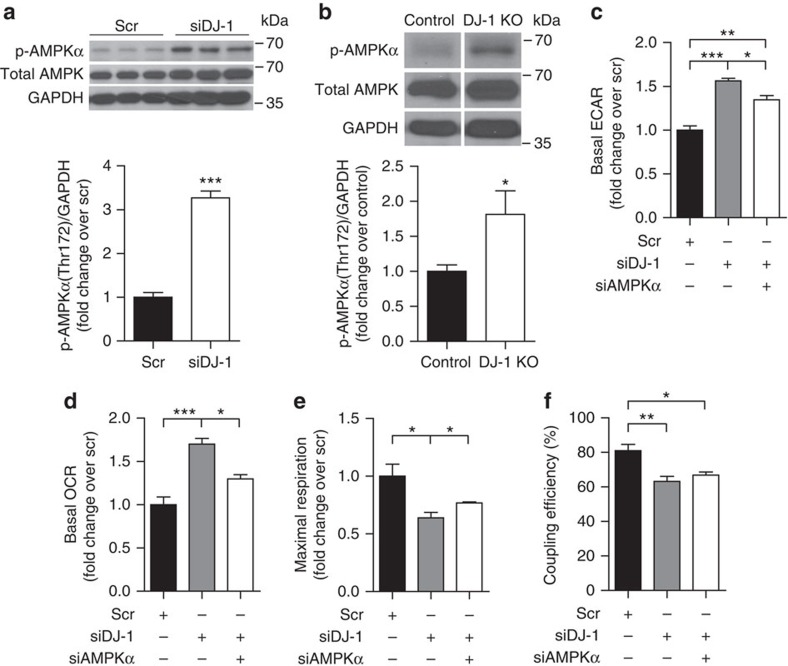
Increased glycolytic activation in DJ-1-deficient cells is dependent on AMPK. (**a,b**) Immunoblot analysis of phospho-AMPKα(Thr172) protein levels in (**a**) C2C12 myotubes (*n*=3 per group) and (**b**) quadriceps lysates from female mice fed a HFD for 3 months from 2 months of age (*n*=6 per group). For **b**, protein bands shown are from non-adjacent lanes on the same gel. Full scan images of immunoblots are shown in Supplementary Fig. 6e,f. (**c**) Basal ECAR, (**d**) basal OCR, (**e**) maximal respiration and (**f**) coupling efficiency measured using the Seahorse flux analyzer in C2C12 cells co-transfected with *Dj1* and *Prkaa2* siRNA (*n*=4 per group). Results are presented as mean±s.e.m. according to the two-tailed unpaired Student's *t*-test for (**a**) and (**b**), and one-way analysis of variance followed by Tukey's *post-hoc* test for **c**–**f**. *P<0.05; **P<0.01; ***P<0.001.

Mitochondrial uncoupling activates AMPK^37^, the master regulator of cellular energy homeostasis, which upregulates glycolysis and enhances oxidative metabolism to maintain cellular energy charge^38^. In keeping with the increased uncoupling observed in *Dj1* knockdown myotubes, we observed an increase in AMPKα phosphorylation in DJ–1-deficient myotubes and muscle tissue (Fig. 5a,b). Co-transfection with AMPKα (*Prkaa2*) siRNA attenuated the increase in ECAR in *Dj1* knockdown cells (Fig. 5c), indicating that AMPK mediates, at least in part, induction of glycolysis in response to increased ROS. AMPKα knockdown also prevented the increase in OCR induced by DJ-1 deficiency (Fig. 5d,e) without restoring coupling efficiency (Fig. 5f), suggesting that AMPK is involved in enhancing mitochondrial oxidation, but not through increased uncoupling.

### DJ-1 deficiency improves glucose homeostasis in mice

**Figure 6:**
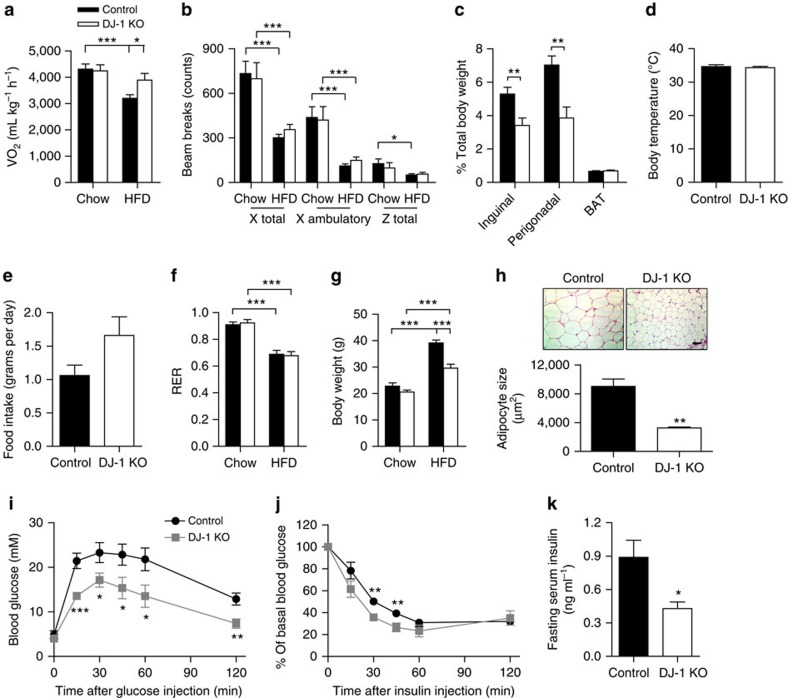
DJ-1 deficiency confers metabolic protection in dietary and genetic models of obesity. (**a**) Oxygen consumption (VO_2_) measured by indirect calorimetry, and (**b**) physical activity in chow- or HFD-fed female mice at 5 months of age (*n*=6 per group for chow-fed mice; *n*=13 per group for HFD-fed mice). HFD feeding was started at 2 months of age. (**c**) Relative weight of inguinal, perigonadal and interscapular brown (BAT) fat pads in HFD-fed female mice (*n*=11 for control and 8 for DJ-1 KO). (**d**) Rectal body temperature in HFD-fed female mice (*n*=6 for control and 4 for DJ-1 KO). (**e**) Food intake in HFD-fed mice, and (**f**) respiratory exchange ratio (RER) (*n*=6 per group for chow-fed mice; *n*=13 per group for HFD-fed mice). (**g**) Body weight in female mice (*n*=6 per group for chow-fed mice; *n*=12 per group for HFD-fed mice). (**h**) Haematoxylin and eosin staining of perigonadal fat sections from HFD-fed female mice and quantification of adipocyte size (*n*=5 per group). Scale bar, 80 μm. (**i**) Glucose tolerance test (GTT) (1 g kg^−1^; *n*=10 per group), (**j**) Insulin tolerance test (ITT) (1.5 U kg^−1^; *n*=12 per group) and (**k**) fasting serum insulin levels (*n*=9 per group) in HFD-fed female mice. Results are presented as mean±s.e.m. according to the two-tailed unpaired Student's *t*-test. *P<0.05; **P<0.01; ***P<0.001.

In line with increased mitochondrial uncoupling, female DJ-1 KO mice, when subject to chronic HFD feeding, showed higher oxygen consumption (VO_2_), indicating increased energy expenditure (Fig. 6a). This difference was not explained by changes in physical activity (Fig. 6b), differences in brown fat mass (Fig. 6c) or brown fat expression of UCP1 that uncouples the respiratory chain for heat generation (Supplementary Fig. 7a). We also found no significant alteration in body temperature, energy intake or fuel source utilized in DJ-1 KO mice (Fig. 6d–f). Accordingly, while chow-fed DJ-1 KO mice did not differ from control littermates in terms of glucose homeostasis up to 5 months of age (Supplementary Fig. 8), when subject to a chronic HFD, DJ-1 KO mice, particularly females, were protected from HFD-induced obesity (Fig. 6g and Supplementary Fig. 9). This resistance to HFD-induced weight gain was primarily due to lower adiposity, as shown by reduced fat accumulation in the inguinal and perigonadal adipose depots (Fig. 6c) and smaller perigonadal adipocytes (Fig. 6h). Consistent with reduced adiposity, DJ-1 KO mice showed significantly lower leptin and resistin concentration in the circulation (Supplementary Table 1). Importantly, increases in adipose tissue mass were not accompanied by changes in expression of adipogenic or lipogenic genes (Supplementary Fig. 7b). Furthermore, the apparent negative energy balance was not accompanied by excess lipid deposition in the liver or changes to hepatic lipid metabolism gene expression (Supplementary Fig. 7c,d). Taken together, the protection from HFD-induced obesity led to enhanced glucose tolerance and insulin sensitivity in female DJ-1 KO mice (Fig. 6i–k and Supplementary Table 1) without changes in insulin signalling in the skeletal muscle (Supplementary Fig. 7e). DJ-1 deficiency also conferred metabolic protection in a genetic model of obesity such that *Dj1*^−/−^*Lep*^*ob/ob*^ mice showed a trend towards reduced body weight and were more glucose tolerant compared with *Dj1*^*+/+*^*Lep*^*ob/ob*^ littermates (Supplementary Fig. 7f,g). These results together indicate an essential contribution of DJ-1 to obesity-associated insulin resistance and glucose intolerance.

### Improved metabolism in DJ-1 KO mice is due to elevated ROS

**Figure 7:**
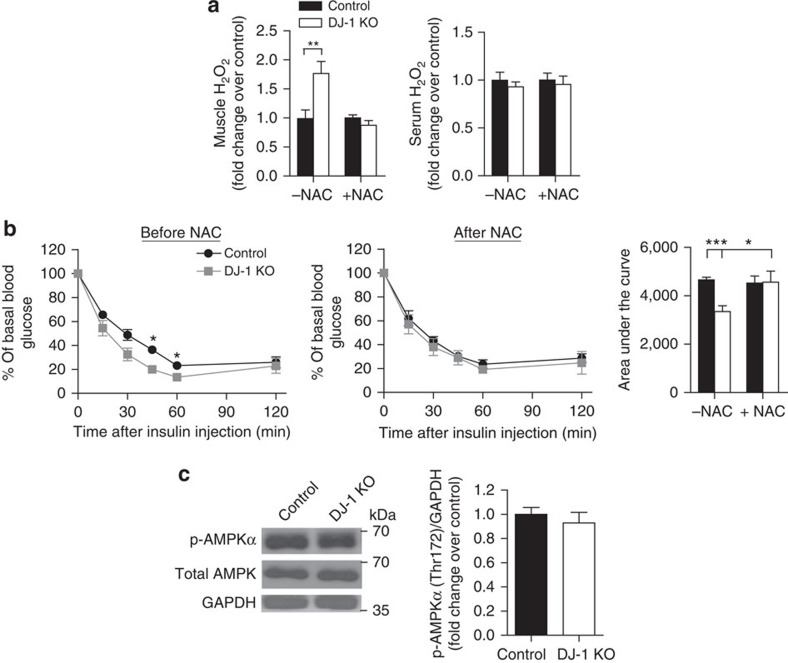
Attenuation of metabolic protection by NAC. Two-month-old female DJ-1 KO mice and littermate controls were fed a HFD for 3 months and administered NAC in drinking water for 7 days (*n*=6 for control and 4 for DJ-1 KO). (**a**) H_2_O_2_ levels measured using the Amplex Red reagent in quadriceps homogenates or serum with and without NAC treatment. (**b**) ITT (1.5 U kg^−1^) and the corresponding area under the curve. (**c**) Immunoblot analysis of phospho-AMPKα(Thr172) protein levels in quadriceps lysates from NAC-treated mice. Full scan images of immunoblots are shown in Supplementary Fig. 9i. Results are presented as mean±s.e.m. according to the two-tailed unpaired Student's *t*-test. *P<0.05; **P<0.01; ***P<0.001.

To confirm an essential contribution of ROS to the observed phenotype of DJ-1 KO mice *in vivo*, we administered a low dose of the antioxidant N-acetyl-L-cysteine (NAC) to HFD-fed female mice. NAC treatment abolished differences in skeletal muscle ROS levels between the genotype groups without affecting circulating ROS (Fig. 7a). Furthermore, NAC abolished the enhanced insulin sensitivity present in DJ-1 KO mice as assessed by insulin tolerance test (ITT) (Fig. 7b). NAC also attenuated the increased AMPKα phosphorylation present in DJ-1 KO mice (Fig. 7c). These results together suggest that elevated muscle ROS were the driving force underlying the enhanced AMPK activation and improved metabolic parameters in the setting of DJ-1 deficiency.

## Discussion

In this work, we have uncovered a novel role of the antioxidant protein DJ-1 in energy homeostasis by regulating metabolic reprogramming in the skeletal muscle via ROS.

**Figure 8:**
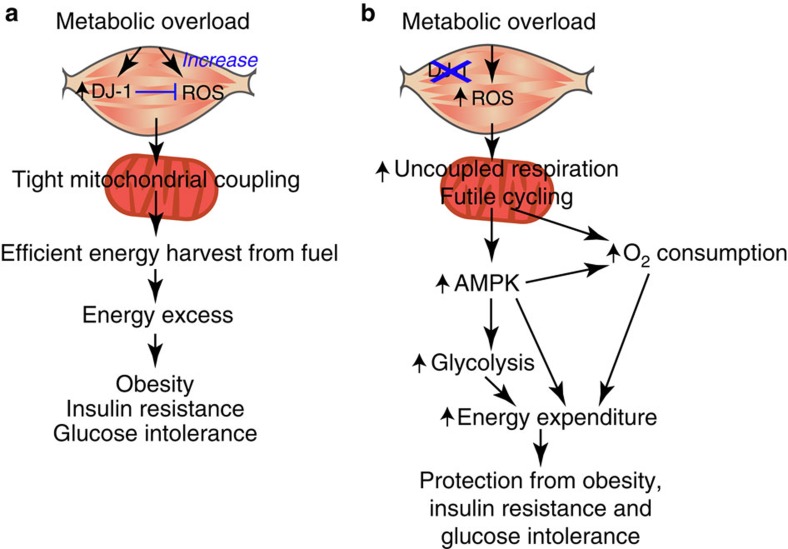
Proposed role of skeletal muscle DJ-1 in metabolic regulation. (**a**) In response to metabolic overload in the skeletal muscle, DJ-1 expression is upregulated. This prevents ROS overproduction and maintains tight coupling of mitochondrial respiration and ATP generation. Consequently, energy is harvested efficiently from nutrient sources, leading to energy excess and development of obesity and related metabolic complications. (**b**) Elevated ROS induced by DJ-1 deficiency increase uncoupled respiration and mitochondrial oxidation, thereby promoting futile cycle activity. This leads to the activation of AMPK, which enhances glycolysis and glucose utilization. These metabolic effects together increase energy expenditure in the skeletal muscle and confer resistance to obesity and type 2 diabetes.

In line with its role as a redox sensor, various oxidative stress conditions have been shown to induce DJ-1 expression^39^. Here we show that HFD, a form of oxidative stress induced by nutritional overload, upregulates *Dj1* and the skeletal muscle seems to be the principal site affected. Given that skeletal muscle is the major tissue responsible for whole-body energy expenditure and insulin-stimulated glucose uptake, induction of DJ-1 might be an evolutionarily conserved adaptive response to offset increased pro-oxidant levels resulting from excessive fuel influx. Our results suggest that DJ-1 acts to maintain efficient fuel utilization; therefore, this upregulation in response to HFD would be evolutionarily advantageous when energy sources are scarce but would be ill-suited in the face of energy excess. Specifically, enhanced production of DJ-1 would function to keep ROS levels low and maintain the tight coupling of mitochondrial respiration and ATP generation. Consequently, energy is harvested efficiently from nutrient sources, predisposing to the development of obesity and metabolic disorders (Fig. 8a). Accordingly, in the absence of DJ-1, HFD-induced overproduction of ROS results in metabolic inefficiency, leading to the activation of AMPK and induction of glycolysis. These metabolic effects together increase energy expenditure in the skeletal muscle and confer resistance to obesity and type 2 diabetes (Fig. 8b).

While extensive evidence suggests that overwhelming production of ROS damages cellular macromolecules and leads to cell senescence or death, it is increasingly apparent that ROS at physiological levels are important in cell signalling and essential for survival. Interventions that extend lifespan such as caloric restriction, mild mitochondrial dysfunction and inhibition of the mammalian target of rapamycin pathway have all been linked to increased ROS production^14^. Furthermore, disruption of antioxidant proteins in rodent models often leads to protection from metabolic stress despite a strong oxidative stress response^40^,^41^. The apparent paradox in the functions of ROS suggests a non-linear dose–response relationship, or hormesis^13^,^14^,^15^.

Our results are in keeping with this hormetic relationship between ROS and health. In particular, the more profound metabolic protection seen in female DJ-1 KO mice may be related to sex differences in intracellular ROS levels. In humans, males express lower levels of antioxidants than female counterparts^42^. As such, females were shown to have lower lipid peroxidation in their skeletal muscle than age-matched males^43^. Consistent with this, in our mouse model, muscle ROS levels were always higher in male mice compared with female counterparts. Therefore, although the higher ROS concentrations in HFD-fed male DJ-1 KO mice were not enough to cause deterioration of glucose metabolism, they may be beyond the optimal level for metabolic benefits.

Our data have revealed a novel mechanism by which ROS activate cellular adaptive pathways to maintain energy homeostasis and confer resistance to metabolic stress. We show that ROS-induced mitochondrial uncoupling and the subsequent metabolic reprogramming with glycolysis induction promote energy expenditure in the skeletal muscle and contribute to healthy adaptation to energy overload. Indeed, emerging evidence supports the therapeutic potential of mild mitochondrial uncouplers for the treatment of obesity and related complications. For example, pharmacologically induced mild mitochondrial uncoupling has recently been shown in proof-of-concept studies to improve glucose homeostasis and attenuate insulin resistance in mice^44^,^45^. These uncouplers appear to act primarily in the liver by promoting lipid oxidation and energy expenditure, whereas our work indicates that mitochondrial uncoupling in the skeletal muscle can also be metabolically beneficial.

Consistent with increased uncoupling, DJ-1 deficiency upregulated muscle expression of *Ucp3* both *in vivo* and *in vitro*. Of note, both UCP2 and UCP3 have been implicated in metabolic homeostasis. UCP2, widely expressed, is proposed to play a role in pancreatic β cell glucose sensing such that its disruption increases β cell ATP levels, thereby enhancing glucose-stimulated insulin secretion^46^. However, the exact physiological role of UCP2 remains controversial^47^. On the other hand, UCP3, predominantly expressed in the skeletal muscle, has been shown to protect against oxidative stress^48^,^49^,^50^. Similar to our DJ-1 KO animals, mice overexpressing *Ucp3* in the skeletal muscle exhibit reduced metabolic efficiency^51^, increased energy expenditure and are protected from HFD-induced obesity and insulin resistance^52^,^53^,^54^. Conversely, obese individuals who are resistant to weight loss therapy demonstrate decreased UCP3 expression accompanied by reduced mitochondrial proton leak in their skeletal muscles^55^, suggesting that increased UCP3 may underlie DJ-1 deficiency-induced metabolic protection in our model.

We show that glycolysis activation in the setting of DJ-1 disruption was dependent on AMPK. Enhanced glycolytic flux would lead to an increase in cytoplasmic ATP production. With no change in mitochondrial oxidative phosphorylation (that is, ATP production from the electron transport chain), total ATP generation would be increased, which may explain the higher ATP concentration observed in DJ–1-deficient myotubes. While higher ATP levels suggest an increase in cellular energy charge, which would not promote AMPK activation, we speculate that in the setting of DJ-1 deficiency AMPK may be activated in response to calcium flux by calcium/calmodulin-dependent protein kinase kinase 2 (CAMKK2)^56^,^57^. Indeed, DJ-1 has been shown to regulate calcium homeostasis in the skeletal muscle such that loss of DJ-1 led to a more than twofold increase in resting intracellular calcium levels^58^.

Taken together, our study highlights the essential role of DJ-1 and ROS in maintaining metabolic homeostasis. Strategies to promote mitochondrial uncoupling and futile cycle activity in skeletal muscle may be a promising therapeutic option for the treatment of obesity and related metabolic disorders.

## Methods

### Animal model and diet

DJ-1 KO mice have been kindly provided by T.W.M.; their generation and genotyping have been previously described^22^. *Dj1*^−/−^*Lep*^*ob/ob*^ mice were generated by breeding *Dj1*^+/−^ mice with *Lep*^*+/ob*^ mice (Jackson Laboratories). All animals were housed in a specific pathogen-free facility at the Toronto Medical Discovery Tower (Toronto, ON, Canada) on a 12:12-h light/dark cycle with free access to water and standard irradiated rodent chow (5% fat; Harlan Teklad). All mice were maintained on a C57BL/6 background. Some mice were fed with a HFD (59% fat, 26% carbohydrates and 15% protein based on caloric content; F3282, Bio-Serv) for 3 months starting at 2 months of age. Animal experiments were approved by the Toronto General Research Institute Animal Care Committee.

### Cell culture

Murine C2C12 myoblasts (CRL-1772; ATCC) were maintained in a growth medium consisting of Dulbecco's modified Eagle's medium (DMEM) (4.5 g l^−1^ D-glucose) supplemented with 10% (vol/vol) FBS (Gibco) and 100 U ml^−1^ penicillin and streptomycin (Gibco). Cells were cultured in a humidified incubator under an atmosphere of 5% CO_2_ at 37 °C. Differentiation was induced by switching to differentiation medium consisting of DMEM supplemented with 2% horse serum (Gibco) when cells reached confluency. Myotubes were used for experiments following 4 days of differentiation. Rat insulinoma cells (INS-1) were cultured in RPMI 1640 medium (Gibco) supplemented with 11.1 mM D-glucose, 2 mM L-glutamine, 10 mM HEPES, 1 mM sodium pyruvate, 50 μM β-mercaptoethanol, 10% (vol/vol) FBS and 100 U ml^−1^ penicillin and streptomycin. Cells were maintained under a humidified condition of 95% air and 5% CO_2_ at 37 °C.

### siRNA transfection

For siRNA transfection, cells cultured in growth medium were transfected with 60 nM Silence Select siRNA (s81228 for *Dj1*; s134961 for *Prkaa2* and s67530 for *Hif1a*; Ambion) using Lipofectamine RNAiMAX reagent (Invitrogen) according to the manufacturer's reverse transfection protocol. For C2C12 cells, 48 h after transfection, cells were switched to differentiation medium and cultured for 96 h or otherwise indicated. For INS-1 cells, 72 h after transfection, cells were collected for subsequent analysis.

### Cell viability

Cell viability was assessed using the MTT (3-(4,5-dimethylthiazol-2-yl)-2,5-diphenyltetrazolium bromide) assay. Briefly, cells were incubated in medium containing 0.1 mg ml^−1^ MTT until dark formazan crystals were visible. Medium was then aspirated, the crystals were solubilized using DMSO and absorbance was measured at a wavelength of 570 nm in a microplate reader. For each experiment, at least four replicates were used.

### Biochemical measurements

Glucose, lactate, pH, glutamine and glutamate levels in the culture medium were measured using the BioProfile FLEX analyzer (NOVA biomedical) at room temperature. Alanine concentration in the culture medium was determined using the L-alanine assay kit (ab83394; Abcam) according to the manufacturer's instructions.

### Cellular metabolic rate

C2C12 myoblasts were seeded at 10,000 cells per well in 24-well XF plates and transfected with scramble or *Dj1* siRNA. Myotubes were generated by replacing the medium with differentiation medium when cells reached confluency. After 4 days of differentiation, myotubes were switched to a bicarbonate-free medium and incubated for 1 h at 37 °C in a non-CO_2_ incubator. A Seahorse XF24 Analyzer (Seahorse Biosciences) was then used to measure the cellular bioenergetic profile. Each cycle included 3 min of mixing, a 2-min wait and measurement over 2 min. Three measurements were obtained at baseline and following injection of oligomycin (1 μM; Sigma), FCCP (0.5 μM; Sigma), and antimycin A and rotenone (1 μM; Sigma). Measurements were normalized to total protein content per well using the Bradford assay.

### Immunofluorescent staining

C2C12 myoblast cells (2.5 × 10^5^) were seeded on sterile glass coverslips and allowed to adhere overnight. Cells were then incubated for 30 min with 100 nM MitoTracker Red CMXRos (M7512) (Invitrogen), a dye that accumulates in active mitochondria, and fixed in 4% paraformaldehyde in 0.1 M PBS (pH 7.4). After washing, cells were incubated with antibody against DJ-1 (D-4; 1:100 dilution; Santa Cruz) for 2 h, after which a FITC-conjugated secondary antibody (1:100 dilution; Jackson ImmunoResearch Laboratories Inc.) was applied. Immunofluorescent images were obtained by a Zeiss inverted fluorescent microscope (Advanced Optical Microscopy Facility, Toronto, Ontario, Canada).

### Microarray

Total RNA from C2C12 myotubes transfected with scramble and *Dj1* siRNA (*n*=3 each) was isolated using the TRIzol reagent (Invitrogen). RNA quality was analysed using the Agilent 2100 BioAnalyzer. About 200 ng of RNA was labelled using Illumina TotalPrep-96 RNA Amplification kit (Ambion) according to the manufacturer's protocol. About 1,500 ng of complementary RNA generated was hybridized onto one Illumina Mouse WG-6 V2 BeadChip by standard procedures. The BeadChip was washed, stained and scanned on the iScan (Illumina). Data were quantified in GenomeStudio Version 2011.1 (Illumina) and analysed using GeneSpring GX12.6 software (Agilent Technologies).

### *In vivo* metabolic analyses

*In vivo* metabolic analyses were performed as previously described^59^,^60^,^61^. Briefly, all overnight fasts were carried out between 1700 and 0900 hours. Fasting blood glucose levels were measured after an overnight fast from tail vein blood with a glucometer (Contour; Bayer HealthCare). For glucose tolerance test, overnight fasted mice were injected i.p. with a D-glucose solution at a dose of 1 g kg^−1^ of body weight. For ITT, mice fasted for 4 h were challenged with an i.p. injection of insulin lispro (Humalog, Lilly) at a dose of 0.75 (for chow-fed mice) or 1.5 U kg^−1^ (for HFD-fed mice). Blood glucose levels were measured from tail vein blood at 0, 15, 30, 45, 60 and 120 min after injection with a glucometer. For insulin signalling experiments, mice fasted overnight were injected i.p. with insulin lispro (5 U kg^−1^, Humalog) or PBS. Tissues were harvested 10 min later. For glucose-stimulated insulin secretion, overnight fasted mice were injected i.p. with glucose (3 g kg^−1^) and blood was collected from saphenous vein at 0, 2, 10 and 30 min after injection. Serum insulin levels were determined by a mouse insulin ELISA kit according to the manufacturer's instructions (Crystal Chem). Body temperature was measured in fed mice between 1000 and 1100 hours using a rectal temperature probe.

### Energy balance measurements

To measure energy expenditure, mice were individually housed in a Comprehensive Laboratory Animal Monitoring System (Columbus Instruments) with free access to food and water. After 24 h acclimatization to the apparatus, data for 48-h measurement were collected. Respiratory exchange ratio was calculated as VCO_2_/VO_2_. Physical activity was determined by infra-red beam breaks in the *x-* and *z*-axes during one measurement interval. Food consumption was determined by weighing the chow or HFD before and after the measurement.

### Analysis of serum parameters

Blood was collected by cardiac puncture into serum separator tubes and centrifuged at 5,000*g* for 10 min at 4 °C. Serum adipokine levels were determined by the Milliplex mouse serum adipokine kit (Millipore).

### ROS determination

To determine ROS levels in cells, cells grown on coverslips or in 96-well plates were washed with antibiotic- and FBS-free DMEM, incubated with 1 μM CM-H_2_DCFDA (C6827; Invitrogen) at 37 °C for 30 min under 5% CO_2_ and washed again with PBS. Fluorescence was visualized using a Zeiss inverted fluorescent microscope or determined using a microplate reader with excitation wavelength at 490 nm and emission wavelength at 520 nm.

For determination of ROS production in skeletal muscle sections, frozen quadriceps cross-sections were washed with ice-cold PBS for 5 min, incubated with 300 nM CM-H_2_DCFDA in PBS at 37 °C for 30 min and washed again in ice-cold PBS to stop the reaction. Slides were mounted with anti-fade mounting media and visualized using a Zeiss inverted fluorescent microscope. Fluorescence intensity of at least four fields per section was quantified using the ImageJ software.

H_2_O_2_ levels in skeletal muscle lysates or serum were determined using the Amplex Red hydrogen peroxide assay kit (Invitrogen) as per the manufacturer's instructions. Briefly, quadriceps muscle was mechanically homogenized in ice-cold lysis buffer (50 mM Tris (pH 7.4), 1% (v/v) Triton X-100, 150 mM NaCl) and centrifuged at 1,000*g* for 10 min at 4 °C. The resulting supernatant was collected for H_2_O_2_ determination. H_2_O_2_ levels were normalized to the corresponding protein concentrations or volume of the serum sample.

### Detection of oxidative stress

Tissue and cellular levels of MDA were determined using a thiobarbituric acid reactive substances (TBARS) assay kit (Caymen Chemical) as per the manufacturer's instructions. Circulating level of GSH to GSSG ratio was determined using a GSH/GSSG ratio detection assay kit (ab138881; Abcam) following the manufacturer's protocol. ATP concentration was measured using the ENLITEN ATP assay system bioluminescence detection kit (Promega).

### Antioxidant treatment

C2C12 myoblasts were transfected with scramble or *Dj1* siRNA and treated with the superoxide dismutase-mimetic and antioxidant tempol (0.1 mM; Sigma). After 48 h, cells were switched to differentiation media and continued to be treated with tempol (0.1 mM) for 96 h.

For *in vivo* antioxidant treatment, female DJ-1 KO mice and littermate controls were fed a HFD for 3 months and administered NAC for 7 days (10 mg l^−1^ in drinking water; Sigma). Body weight and ITT were determined before and after treatment.

### Histology

Perigonadal adipose tissue was harvested, fixed in 4% paraformaldehyde in 0.1 M PBS (pH 7.4) and processed to paraffin blocks. Slides were cut in 7-μm-thick sections with 150 μm separation on three levels. Adipose tissue sections were stained with haematoxylin and eosin. Adipocyte size was measured using the cellSens software (Olympus). For Oil-red-O staining, livers were isolated, immersed in Tissue-Tek O.C.T. compound (Sakura) and immediately frozen using liquid nitrogen. Sections were fixed with 4% paraformaldehyde, washed with 60% isopropanol, stained with Oil-red-O (Sigma), then washed again with 60% isopropanol.

### Fibre type distribution analysis

To determine fibre type distribution in soleus muscle from HFD-fed female mice, immunofluorescence analysis of myosin heavy chain expression was performed as described previously^62^. All fibres within the entire muscle cross-section were characterized.

### MtDNA analysis

Total DNA was extracted from quadriceps muscle using phenol/chloroform/isoamyl alcohol (25:24:1). MtDNA content was calculated using real-time quantitative PCR by measuring expression level of a mitochondrial-encoded gene (*Cox2*) and normalizing it to expression level of a nuclear-encoded gene (Cyclophilin A (*Ppia*)). Primer sequences are listed in Supplementary Table 2.

### Transmission electron microscopy

Quadriceps muscle was cut into small pieces and fixed in Karnovsky's style fixative overnight at 4 °C. Samples were then postfixed in 1% osmium tetroxide in Sorensen's phosphate buffer for 2 h at room temperature, dehydrated and embedded in Epon Araldite. Resin blocks containing the tissue samples were cut using a Reichert Ultracut E microtome, and ultrathin sections (60–90 nm) were mounted on 300-mesh copper grids, counter stained using saturated uranyl acetate and lead citrate, and imaged by a Hitachi H7000 transmission electron microscope at an accelerating voltage of 75 kV.

### Immunoblotting

INS-1 cells, C2C12 myotubes, skeletal muscle and interscapular brown fat tissue were mechanically homogenized in ice-cold lysis buffer and centrifuged at 14,000*g* for 10 min at 4 °C. The resulting supernatant was separated by SDS–PAGE, and immunoblotted with antibodies to sarcomeric myosin heavy chain (MF20; 1:1,000 dilution; Developmental Studies Hybridoma Bank, University of Iowa), DJ-1 (D-4; 1:500 dilution), UCP1 (M-17; 1:500 dilution), actin (C-2; 1:1,000 dilution) (Santa Cruz Biotechnology), phospho-AMPKα (Thr172) (40H9; 1:1,000 dilution), total AMPKα (23A3; 1:1,000 dilution), phospho-Akt (Ser473) (1:500 dilution), total Akt (1;1,000 dilution), Bcl-xL (H-5; 1:500 dilution), GAPDH (14C10; 1:5,000 dilution) or α/β-tubulin (1:1,000 dilution) (Cell Signalling Technology). Protein band intensity was quantified by ImageJ software.

### Total RNA isolation and quantitative RT–PCR

Total RNA from liver tissue was isolated with RNeasy Mini Kit (Qiagen) according to the manufacturer's protocol. Total RNA from C2C12 myotubes, skeletal muscle and perigonadal adipose tissue was isolated using the TRIzol reagent. mRNA was reverse transcribed with random primers using the M-MLV enzyme (Invitrogen), and quantitative PCR was performed using specific primers and SYBR Green master mix (Applied Biosystems) on a 7900HT Fast Real-Time PCR System (Applied Biosystems). Primer sequences are listed in Supplementary Table 2. Each sample was run in triplicate. The relative mRNA abundance of each gene was normalized to the expression levels of the housekeeping gene *18S*.

### Statistical analysis

The sample size was estimated to be 10–15 per genotype for metabolic studies and 3–6 for *in vitro* experiments and gene and protein expressions, as has been demonstrated by previous publications to be adequate for cell line and animal studies. Data are presented as mean±s.e.m. Values were analysed by two-tailed unpaired Student's *t*-test or one-way analysis of variance followed by Tukey's *post-hoc* test, as appropriate, using GraphPad Prism version 5. *P* values<0.05 were accepted as statistically significant.

## Additional information

**Accession codes:** Microarray data have been deposited in NCBI's Gene Expression Omnibus (GEO) under accession code GSE63051.

**How to cite this article**: Shi, S. Y. *et al.* DJ-1 links muscle ROS production with metabolic reprogramming and systemic energy homeostasis in mice. *Nat. Commun.* 6:7415 doi: 10.1038/ncomms8415 (2015).

## Supplementary Material

Supplementary Information Supplementary Figures 1-9 and Supplementary Tables 1-2
