# Supplementary material for: DJ-1 links muscle ROS production with metabolic reprogramming and systemic energy homeostasis in mice
Source: Nat Commun. 2015 Jun 16;6:7415. doi: 10.1038/ncomms8415 (PMC4490365; doi:10.1038/ncomms8415)
Supplement: Supplementary Information — Supplementary Figures 1-9 and Supplementary Tables 1-2 [file ncomms8415-s1.pdf]

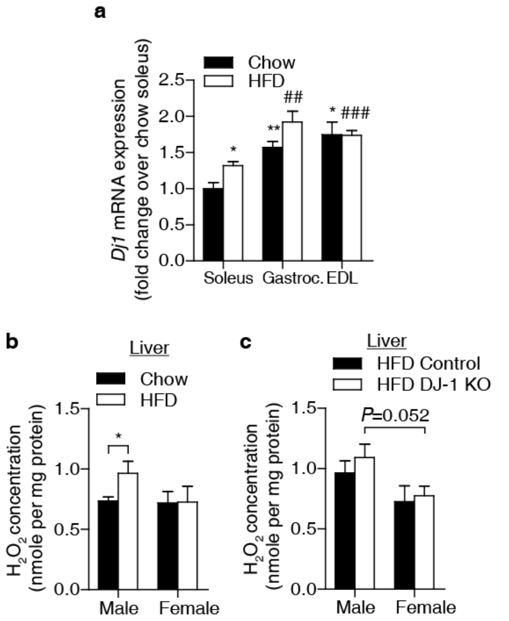

Supplementary Figure 1. DJ-1 modulates ROS concentration in mouse skeletal muscle.

(**a**) mRNA levels of *Dj1* measured by quantitative RT-PCR in *soleus*, *gastrocnemius* (Gastroc.) and *extensor digitorum longus* (EDL) muscle from C57BL/6 mice maintained on standard chow or fed a HFD for 3 months starting at 2 months of age (n=3-6 per group). * and # denote significant differences from the chow and HFD soleus group, respectively. (**b-c**) H_2O_2 levels measured using the Amplex Red reagent in liver tissue in (**b**) chow and HFD-fed C57BL/6 mice and (**c**) HFD-fed DJ-1 KO mice (n=5 per group). Data are normalized to sample protein content. Results are presented as mean ± s.e.m. according to the two-tailed unpaired Student's *t* test. *$P<0.05$; ** or ##$P<0.01$; ###$P<0.001$.

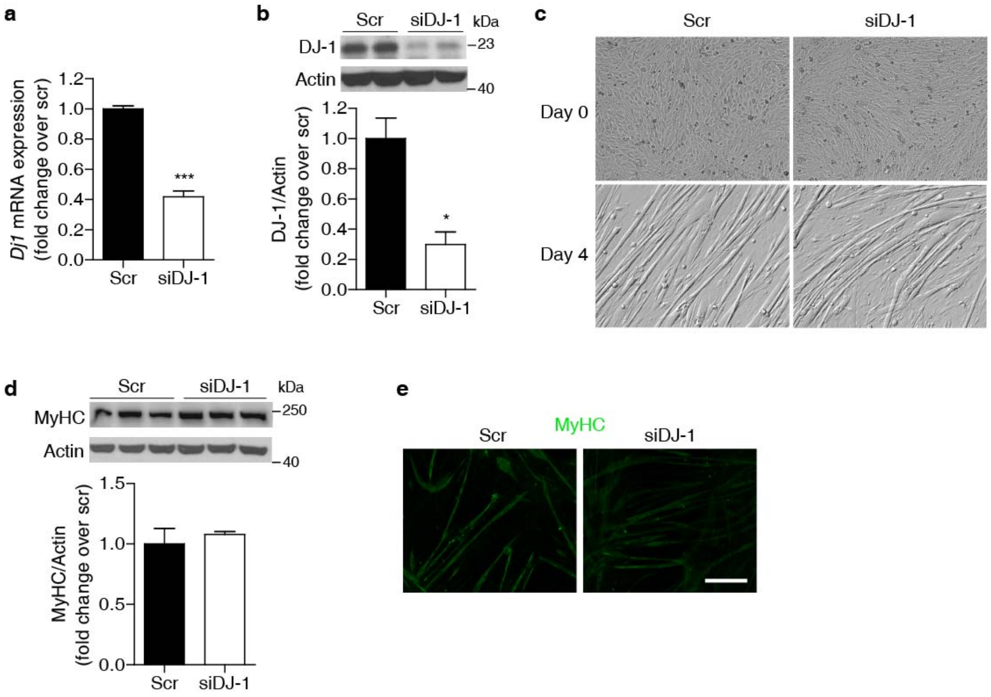

Supplementary Figure 2. Knockdown of DJ-1 in C2C12 cells elevates intracellular ROS levels.

(**a**) mRNA expression of *Dj1* in C2C12 myotubes after *Dj1* knockdown measured by quantitative RT-PCR. Results are normalized to *18S* expression (n=6 per group). (**b**) Immunoblot analysis of DJ-1 protein levels in C2C12 myotubes after *Dj1* knockdown (n=3 per group). Protein band intensity was quantified by ImageJ software. (**c**) Representative phase contrast micrographs of C2C12 myotubes at day 0 and day 4 after differentiation induction. Original magnification, 10×. (**d**) Immunoblot analysis of myosin heavy chain (MyHC) protein levels in C2C12 myotubes (n=3 per group). Protein band intensity was quantified by ImageJ software. (**e**) Representative micrographs of MyHC immunofluorescence staining in C2C12 myotubes after *Dj1* knockdown. Scale bar, 80 µm. Results are presented as mean ± s.e.m. according to the two-tailed unpaired Student's *t* test. *$P < 0.05$; ***$P < 0.001$.

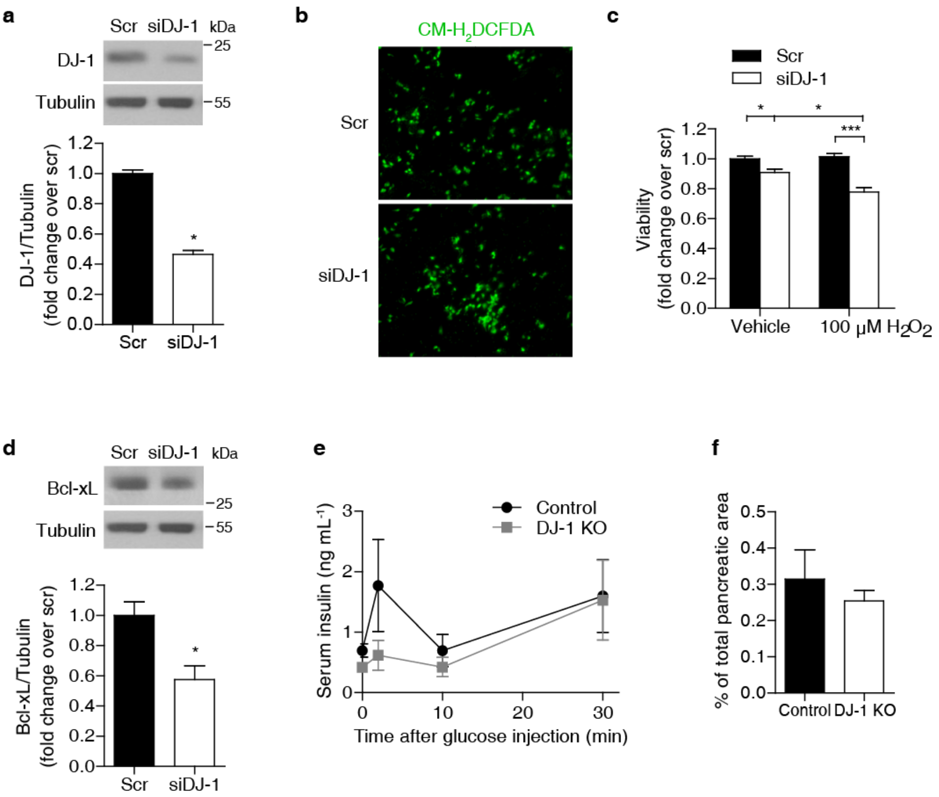

Supplementary Figure 3. DJ-1 protects pancreatic β-cells from oxidative stress-induced cell death.

(**a-d**) INS-1 832/13 cells were transfected with scramble or *Dj1* siRNA, and cells were harvested 72 hours later for analysis. (**a**) Immunoblot analysis of DJ-1 protein levels in INS-1 cells (n=3 per group). (**b**) Representative micrographs showing ROS levels assessed using CM-H_2DCFDA in INS-1 cells. Original magnification, 10×. (**c**) 48 hours post-transfection, INS-1 cells were treated with 100 μM H_2O_2 for 24 hours, after which cell survival was assessed using an MTT assay (n=4 per group). Results are expressed as fold change relative to the scramble group. (**d**) Immunoblot analysis of Bcl-xL protein levels in INS-1 cells (n=3 per group). (**e**) Serum insulin levels in response to an intraperitoneal injection of glucose (3 g/kg) in female mice fed a HFD for 3 months starting at 2 months of age (n=5 per group). (**f**) Quantification of β-cell area from pancreatic sections immunostained for insulin in HFD-fed female mice (n=3-4 per group). β-cell area is expressed as percent of total pancreatic area. Results are presented as mean ± s.e.m. according to the two-tailed unpaired Student's t test. *$P < 0.05$; ***$P < 0.001$.

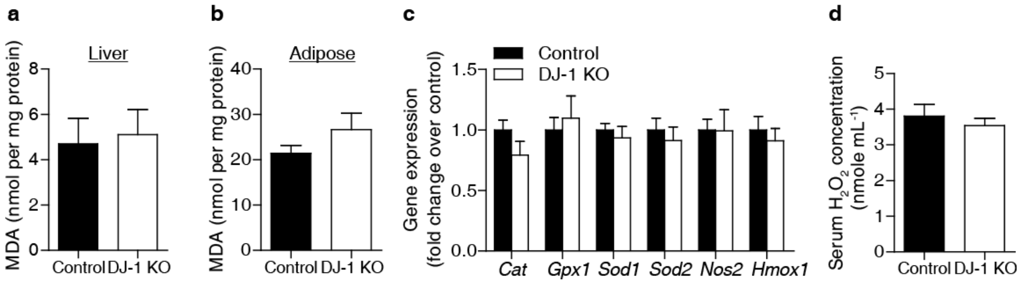

Supplementary Figure 4. DJ-1 deficiency does not induce overt oxidative stress.

(**a-b**) Malondialdehyde (MDA) levels measured using a TBARS assay kit in (**a**) liver and (**b**) perigonadal adipose tissue from female mice fed a HFD for 3 months starting at 2 months of age. Results are normalized to sample protein content (n=4 per group). (**c**) mRNA expression of genes involved in oxidative stress response measured by quantitative RT-PCR in *quadriceps* tissue from HFD-fed female mice (n=8 for control and 9 for DJ-1 KO). (**d**) H_2O_2 concentration measured using the Amplex Red reagent in serum from HFD-fed female mice (n=10 per group). Results are presented as mean ± s.e.m. according to the two-tailed unpaired Student's *t* test.

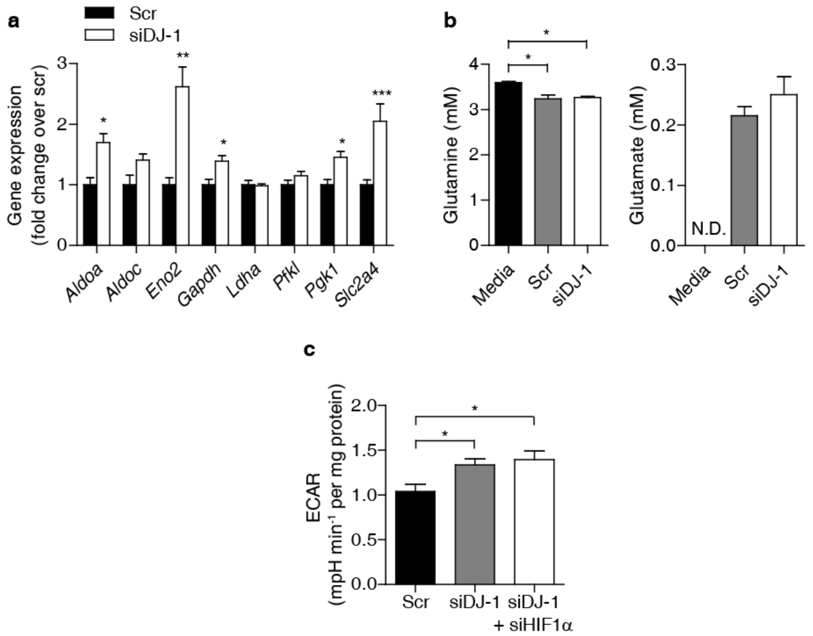

Supplementary Figure 5. DJ-1 deficiency induces glycolysis activation in muscle cells.

(**a**) mRNA expression of glycolytic genes measured by quantitative RT-PCR in C2C12 myotubes (n=3-6 per group). (**b**) Glutamine and glutamate concentration in conditioned media from C2C12 myotubes (n=3 per group). N.D., not detected. (**c**) Basal ECAR measured using the Seahorse flux analyzer in C2C12 cells co-transfected with *Dj1* and *Hif1a* siRNA (n=4 per group). Results are presented as mean ± s.e.m. according to the two-tailed unpaired Student's *t* test for (**a**), and one-way ANOVA followed by Tukey's post hoc test for (**b**)-(**c**). **P* < 0.05; ***P*<0.01; ****P*<0.001.

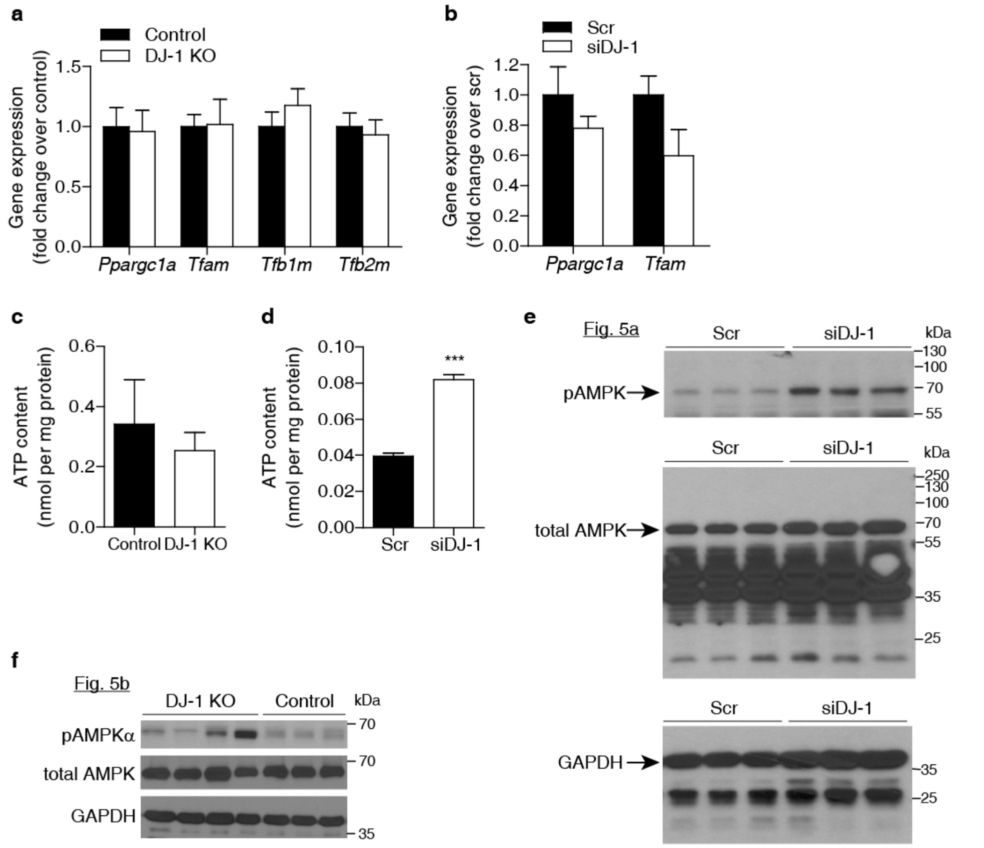

Supplementary Figure 6. DJ-1 deficiency has no effect on mitochondrial biogenesis or morphology.

(**a-b**) mRNA expression of genes involved in mitochondrial biogenesis measured by quantitative RT-PCR in (**a**) *quadriceps* tissue from female mice fed a HFD for 3 months starting at 2 months of age (n=5-9 per group) and (**b**) C2C12 myotubes (n=3 per group). (**c-d**) ATP content in (**c**) *quadriceps* tissue from HFD-fed female mice (n=4 per group) and (**d**) C2C12 myotubes (n=3 per group). Results are normalized to sample protein content. (**e-f**) Full scans of immunoblots presented in Fig. 5a-b. Results are presented as mean ± s.e.m. according to the two-tailed unpaired Student's *t* test. ***P<0.001.

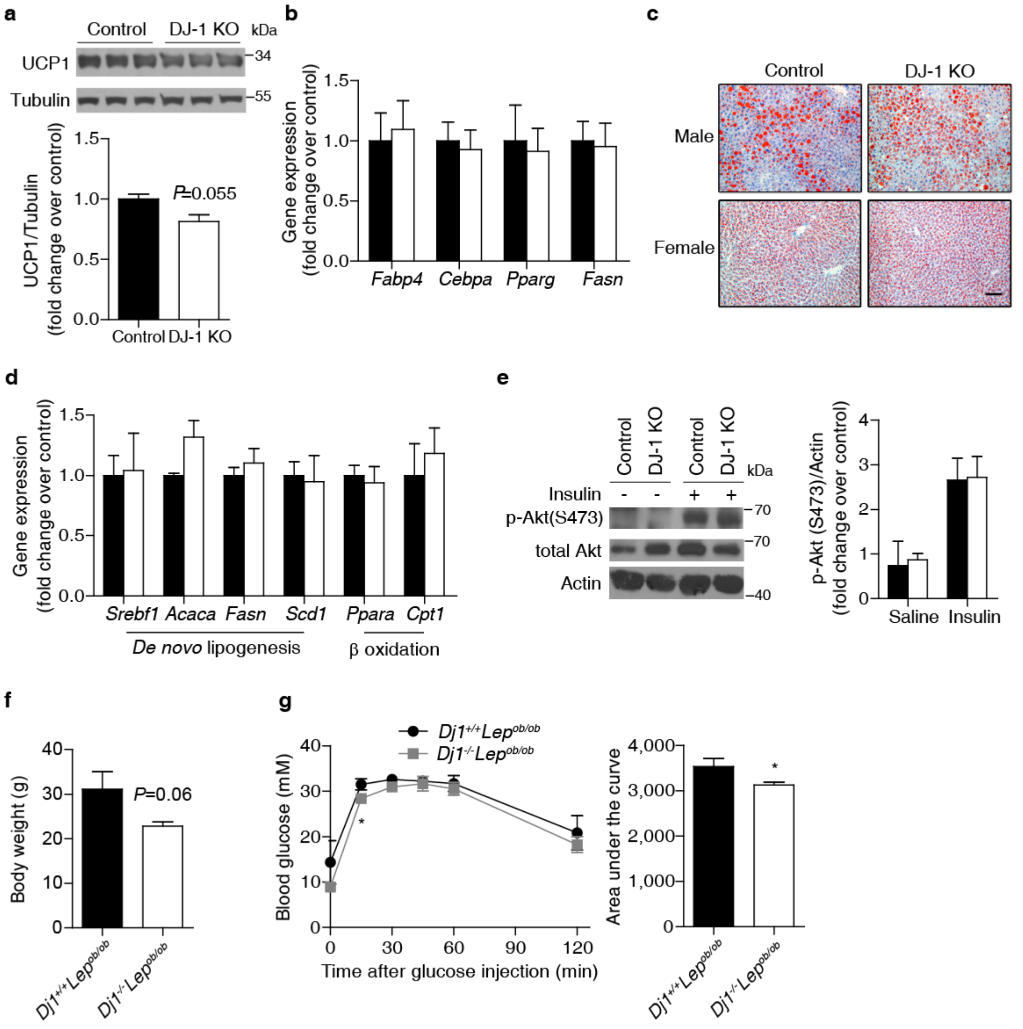

Supplementary Figure 7. Female DJ-1 KO mice are protected from diet-induced obesity and glucose intolerance.

(**a**) Immunoblot analysis of UCP1 protein levels in interscapular brown adipose tissue lysates from female mice fed a HFD for 3 months starting at 2 months of age (n=3 per group). (**b**) mRNA expression of genes involved in adipogenesis and lipogenesis measured by quantitative RT-PCR in perigonadal adipose tissue from HFD-fed mice (n=5-6 per group). (**c**) Representative micrographs of Oil-red-O staining of liver sections from HFD-fed mice. Scale bar, 80 µm. (**d**) mRNA expression of genes involved in lipid metabolism measured by quantitative RT-PCR in liver tissue from HFD-fed mice (n=3-6 per group). (**e**) Mice fasted overnight were injected with insulin (5 U/kg, i.p.) or PBS, and *gastrocnemius* was harvested 10 min later and processed for immunoblotting for p-Akt(S473) (n=3 per group). (**f**) Body weight (n=3-4 per group) and (**g**) GTT (1 g/kg; n=5-6 per group) in chow-fed *Dj1*$^{-/-}$*Lep*$^{ob/ob}$ and *Dj1*$^{+/+}$*Lep*$^{ob/ob}$ mice at 6 weeks of age. Results represent mean ± s.e.m. according to the two-tailed unpaired Student's *t* test. **P*<0.05.

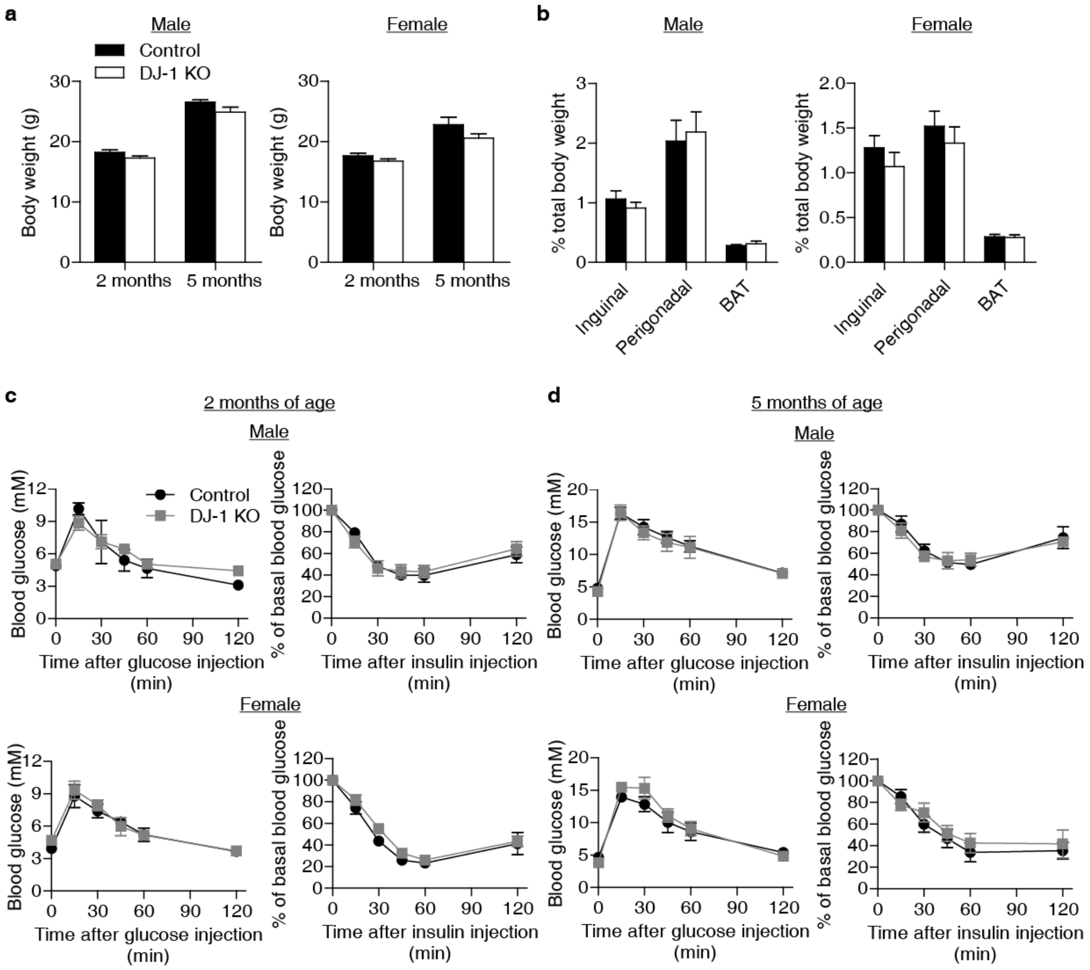

Supplementary Figure 8. No apparent metabolic disturbances in DJ-1 KO mice under standard chow-fed conditions.

(**a**) Body weight measured at 2 and 5 months of age in chow-fed mice (n=12-17 per group for results at 2 months of age; n=5-7 per group for results at 5 months of age). (**b**) Inguinal, perigonadal and interscapular brown fat pads were harvested from 5-month-old chow-fed mice and weighed (n=7-9 per group). Results are expressed relative to total body weight. BAT, brown adipose tissue. (**c-d**) GTT (1 g/kg) and ITT (0.75 U/kg) in chow-fed DJ-1 KO mice and littermate controls at (**c**) two (n=4-8 per group) and (**d**) five months of age (n=6-8 per group). Results represent mean ± s.e.m. according to the two-tailed unpaired Student's *t* test.

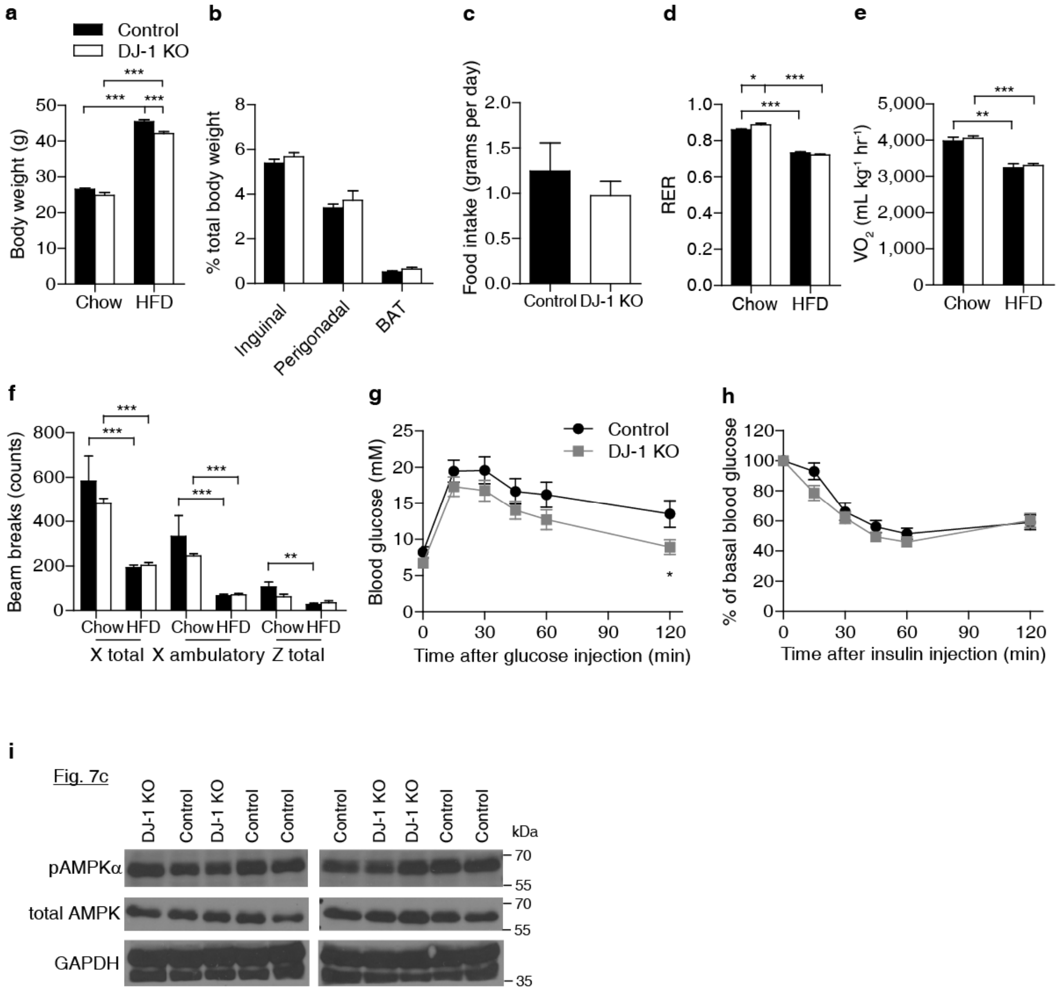

Supplementary Figure 9. Energy balance and glucose metabolism parameters in male mice.

(**a**) Body weight (n=6-8 per group) in male chow- and HFD-fed mice at 5 months of age. HFD was started at 2 months of age. (**b**) Relative weight of inguinal, perigonadal and interscapular brown (BAT) fat pads in male HFD-fed mice (n=8 per group). (**c-f**) Male mice fed a standard chow (n=4 per group) or HFD (n=8 per group) were housed individually in metabolic chambers with free access to food and water and energy balance data were collected for 48 hr. (**c**) Daily food intake in HFD-fed mice; (**d**) respiratory exchange ratio (RER); (**e**) oxygen consumption (VO_2); and (**f**) physical activity. (**g**) GTT (1 g/kg; n=13-14 per group); and (**h**) ITT (1.5 U/kg; n=12 per group) in male HFD-fed mice. (**i**) Full scans of immunoblots presented in Fig. 7c. Results are mean ± s.e.m. according to the two-tailed unpaired Student's *t* test. **P*<0.05; ***P*<0.01; ****P*<0.001.

Supplementary Table 1: Circulating parameters in mice fed a HFD for 3 months from 2 months of age.

	**Male**		**Female**	
	Control	DJ-1 KO	Control	DJ-1 KO
Random blood glucose (mM) (n≥5)	9.79±0.43	9.66±0.68	7.69±0.36	7.73±0.40
Fasting blood glucose (mM) (n≥8)	8.21±0.74	6.72±0.72	4.89±2.42	4.31±1.72
Serum TNF-α (pg mL^{-1}) (n≥4)	4.49±1.18	2.56±0.39	1.98±0.31	2.74±0.60
Serum IL-6 (pg mL^{-1}) (n≥4)	11.00±3.55	4.92±0.61	6.38±1.69	4.99±0.61
Serum leptin (ng mL^{-1}) (n≥4)	31.37±15.13	36.24±7.91	41.47±3.36	8.87±2.82***
Serum resistin (ng mL^{-1}) (n≥4)	0.62±0.12	1.02±0.12	1.84±0.18	0.76±0.08**
Serum total PAI-1[†] (ng mL^{-1}) (n≥4)	3.20±0.88	2.14±0.71	2.43±0.50	1.68±0.73
Serum MCP-1 (pg mL^{-1}) (n≥4)	13.06±5.60	14.05±4.16	18.79±5.78	13.49±5.69

Results are presented as mean ± s.e.m. according to the two-tailed unpaired Student's *t* test. ***P* < 0.01; ****P* < 0.001 compared to the respective controls.
[†]PAI-1, plasminogen activator inhibitor 1; MCP-1, monocyte chemoattractant protein 1.

Supplementary Table 2: Primer sequences for quantitative RT-PCR

Gene	Forward (5'-3')	Reverse (5'-3')
18s	AGTCCCTGCCCTTTGTACACA	CGATCCGAGGGCCTCACTA
Acaca	CTCCAGGACAGCACAGATCA	TGACTGCCGAAACATCTCTG
Aldoa	AGCTCCTTCTTCTGCTCCG	TTAGTCCTTTCGCCTACCCA
Cebpa	AAGAACAGCAACGAGTACCGG	CATTGTCACTGGTCAGCTCCA
Cat1	ATGGCTTTTGACCCAAGCAA	CGGCCCTGAAGCTTTTTGT
Cox2	CCATAGGGCACCAATGATACTG	AGTCGGCCTGGGATGGCATC
Cpt1	GCAGAGCACGGCAAAATGA	CTTTCGACCCGAGAAGACCTT
Dj1	ATCTGAGTCGCCTATGGTGAAG	ACCTACTTCGTGAGCCAACAG
Fabp4	GACGACAGGAAGGTGAAGAG	ACATTCCACCACCAGCTTGT
Fasn	TGGGTTCTAGCCAGCAGAGT	ACCACCAGAGACCGTTATGC
Eno2	CACATCCATACCGATCACCA	CCCCAATATCCTGGAGAACA
Gapdh	TCACCACCATGGAGAAGGC	GCTAAGCAGTTGGTGGTGCA
Gpx1	GCGGCCCTGGCATTG	GGACCAGCGCCCATCTG
Hk2	GGGCATGAAGGGCGTGTCCC	TCTTCACCCTCGCAGCCGGA
Hmox1	GCCACCAAGGAGGTACACAT	GCTTGTTGCGCTCTATCTCC
Il6	CTCTGGGAAATCGTGGAAATG	AAGTGCATCATCGTTGTTCATACA
Ldha	GCAACATTCACACCACTCCA	TCCGTTACCTGATGGGAGAG
Nos2	CCTGGTACGGGCATTGCT	GCTCATGCGGCCTCCTTT
Pfkl	GATGAGGAAGACTTTGGCCC	CTACCGTGGACCTGGAGAAA
Pgk1	CAGCCTTGATCCTTTGGTTG	CTGACTTTGGACAAGCTGGA
Ppara	CAGGGTACCACTACCGAGTTCAC	CCGAATAGTTCGCCGAAAGA
Pparg	GCCCTTTGGTGACTTTATGG	CAGCAGGTTGTCTTGGATGT
Ppargc1a	AACCACACCCACAGGATCAGA	TCTTCGCTTTATTGCTCCATGA
Ppia	ACACGCCATAATGGCACTGG	CAGTCTTGGCAGTGCAGAT
Scd1	GCGATACACTCTGGTGCTCA	CCCAGGGAAACCAGGATATT
Slc2a4	TCATTGTCGGCATGGGTTT	GGCAAATAGAAGGAAGACGTAAGG
Sod1	ACCAGTGCAGGACCTCATTTTAA	TCTCCAACATGCCTCTCTTCATC
Sod2	CACATTAACGCGCAGATCATG	CCAGAGCCTCGTGGTACTTCTC
Srebf1	GATCAAAGAGGAGCCAGTGC	TAGATGGTGGCTGCTGAGTG
Tfam	GCACCCTGCAGAGTGTTCAA	CGCCCAGGCCTCTACCTT
Tfb1m	TGCGTTTCAGTTTCGAAGGA	TCGAGGCGTTGTGCTTCAG
Tfb2m	TTTCCACTTGGTAAAGCATTGCT	GATCAACCGTACTCAGTGAACGTAA
Tnf	GAACTGGCAGAAGAGGCACT	AGGGTCTGGGCCATAGAACT
Ucp1	GTGAAGGTCAGAATGCAAGC	AGGGCCCCCTTCATGAGGTC
Ucp2	TCCACGCAGCCTCTACAAT	GACCTTTACCACATCTGTAGGC
Ucp3	CAGAGGGACTATGGATGCCTAC	AGGTGAGACTCCAGCAACTTCT
